# Assessment of the *In Vivo* Genotoxicity of New Lead Compounds to Treat Sickle Cell Disease

**DOI:** 10.3390/molecules16042982

**Published:** 2011-04-06

**Authors:** Jean Leandro dos Santos, Priscila Longhin Bosquesi, Eliana Aparecida Varanda, Lídia Moreira Lima, Man Chin Chung

**Affiliations:** 1 Laboratório de Pesquisa e Desenvolvimento de Fármacos (Lapdesf), Departamento de Fármacos e Medicamentos, Faculdade de Ciências Farmacêuticas, Univ Estadual Paulista (UNESP), Rodovia Araraquara Jaú Km. 01, 14801-902, Araraquara, SP, Brazil; 2 Departamento de Ciências Biológicas, Faculdade de Ciências Farmacêuticas, Univ Estadual Paulista (UNESP), Rodovia Araraquara Jaú Km. 01, 14801-902, Araraquara, SP, Brazil; 3 Laboratório de Avaliação e Síntese de Substâncias Bioativas (LASSBio), Faculdade de Farmácia, Univ Federal do Rio de Janeiro (UFRJ), Centro de Ciências da Saúde, Cidade Universitária, Ilha do Fundão, 21.944-190–Rio de Janeiro, RJ, Brazil

**Keywords:** genotoxicity assay, micronucleus, sickle cell, phthalimide derivatives

## Abstract

The compounds 1,3-dioxo-1,3-dihydro-2H-isoindol-2-yl)methyl nitrate (**C1**), (1,3-dioxo-1,3-dihydro-2*H*-isoindol-2-yl)ethyl nitrate (**C2**), 3-(1,3-dioxo-1,3-dihydro-2*H*-isoindol-2-yl)benzyl nitrate (**C3**), 4-(1,3-dioxo-1,3-dihydro-2*H*-isoindol-2-yl)-*N*-hydroxy-benzenesulfonamide (**C4**), 4-(1,3-dioxo-1,3-dihydro-2*H*-isoindol-2-yl)benzyl nitrate (**C5**), and 2-[4-(1,3-dioxo-1,3-dihydro-2*H*-isoindol-2-yl)phenyl]ethyl nitrate (**C6**) were evaluated with a micronucleus test using mouse peripheral blood to identify new candidate drugs for the treatment of sickle cell disease (SCD) that are safer than hydroxyurea. The compounds induced an average frequency of micronucleated reticulocytes (MNRET) of less than six per 1,000 cells at 12.5, 25, 50, and 100 mg/kg, whereas hydroxyurea induced an average MNRET frequency of 7.8, 9.8, 15, and 33.7 per 1000 cells respectively, at the same concentrations. Compounds **C1–C6** are new non-genotoxic *in vivo* candidate drugs for the treatment of SCD symptoms.

## 1. Introduction 

Sickle cell disease (SCD), one of the most prevalent haematological diseases in the World, is an autosomal recessive disorder characterized by a point mutation (GTG to GAG) in the sixth codon of the β-globin gene, which leads to the substitution of a glutamic acid for a valine residue in the β-globin chain. This substitution has profound consequences for the structure of haemoglobin (Hb). In conditions of reduced oxygen tension, the exposed non-polar regions of the Hb interact with each other through the β chain subunits, forming a relatively insoluble polymer, which can deform the erythrocyte cytoskeleton or damage the structure of the membrane [1].

Currently, hydroxyurea (HU) is the main drug available for the treatment of sickle cell anaemia that is authorized by the U.S. Food and Drug Administration. HU is a selective inhibitor of ribonucleotide diphosphate reductase, an enzyme that converts ribonucleotides to deoxy-ribonucleotide diphosphates, preventing cells from leaving the G1/S phase of the cell cycle [2]. The beneficial effects of HU in the treatment of SCD are associated, in part, with its ability to be bioconvert *in vivo* to nitric oxide (NO). NO contributes significantly to vaso-occlusive disorders, reducing the expression of adhesion molecules and inhibiting platelet aggregation, thus maintaining the vascular homeostatic balance [3]. It has also been proposed that NO production by HU stimulates foetal haemoglobin (HbF) production via the soluble guanylate cyclase pathway [4].

Despite these beneficial effects, HU also has adverse/deleterious effects, such as myelosuppression, pro-inflammatory activity, and in some cases, development of cancer [5]. Short- or mid-term tolerance of HU treatment has also been reported, insofar as 25% of patients do not respond to HU. In many cases, the beneficial effects of chronic HU treatment are reduced or even abolished [6]. Therefore, the introduction of new drugs that combine the beneficial effects of HU without potential toxicity is essential.

**Figure 1:**
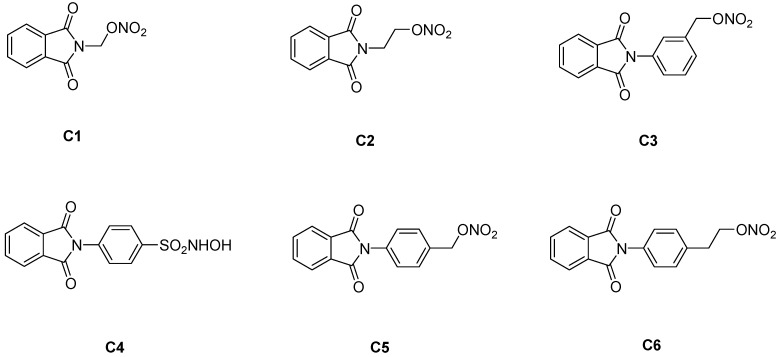
Chemical structures of compounds **C1-C6**.

Using molecular modification, Santos and co-workers have developed new organic nitrate esters **C1–C6** with NO-donor properties (Figure 1) [7]. These compounds were designed to offer dual mechanisms of action: the inhibition of tumor necrosis factor α (TNFα) and nitric oxide generation. All the compounds synthesized demonstrated anti-inflammatory and analgesic effects greater than those of drugs like indomethacin and dipyrone. These compounds also reduced leukocyte infiltration and they were able to generate nitric oxide. *In vitro* studies have also shown that the compounds increase gamma globin expression to higher levels than does HU [8]. *In vivo* studies have demonstrated that these compounds increase HbF levels and modulate pro-inflammatory cytokines, such as TNFα and interleukin 1. Interestingly, the compounds were less genotoxic than HU to prokaryotic cells in a Salmonella/microsome assay [9].

To identify new, safer candidate drugs for the treatment of SCD, we assessed the *in vivo* genotoxicity of these organic nitrate esters using a micronucleus test with mouse peripheral blood. Among the mutagenicity tests advocated by international agencies and governmental institutions, the micronucleus test using the peripheral blood of rodents *in vivo* is widely accepted and recommended for the evaluation and registration of the new chemicals and pharmaceuticals that annually enter the world market.

Genetic toxicology assays, in combination with pharmacological characterization, can identify more adequately those candidate drugs that warrant further development with clinical trials.

## 2. Results and Discussion 

**Figure 2:**
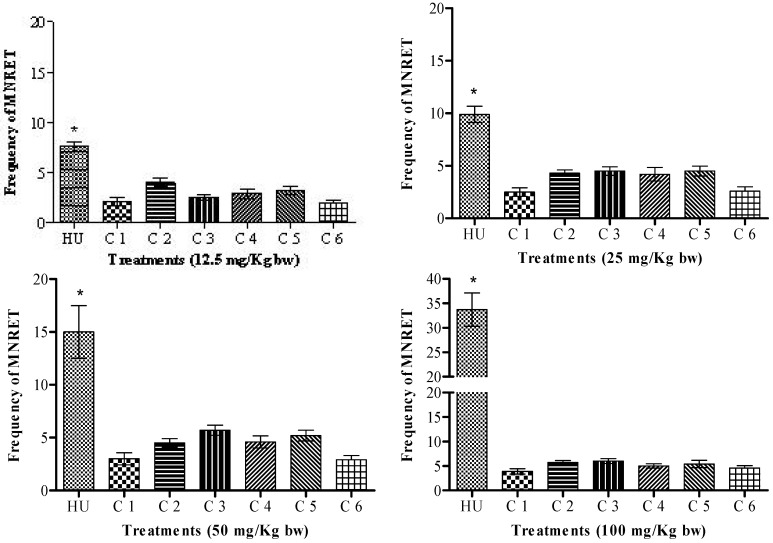
Average frequency of micronucleated reticulocytes (MNRET) and standard deviation of 1,000 cells from mice treated with the drug hydroxyurea (HU) and all the synthesized compounds **C1-C6** at different concentrations. *P < 0.05.

**Figure 3:**
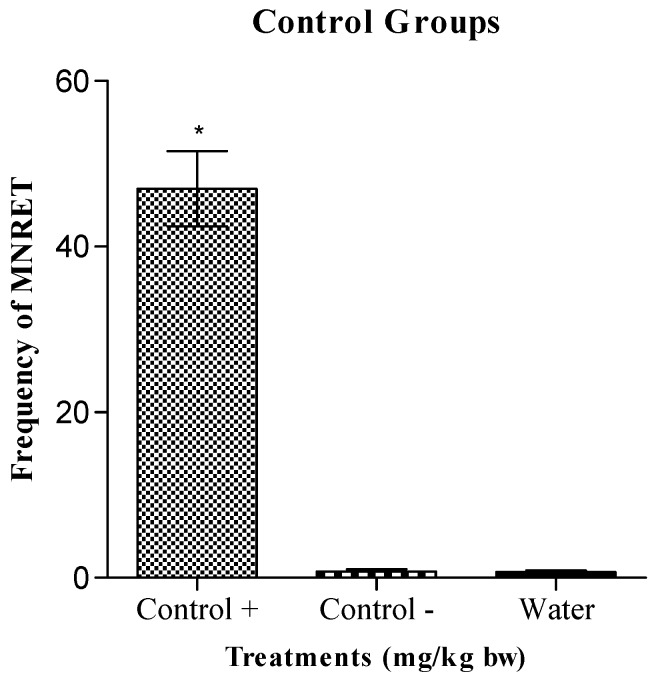
Average frequency of micronucleated reticulocytes (MNRET) and standard deviation of 1000 cells from mice treated with the positive control cyclophosphamide (100 mg/Kg), CMC / Tween (negative control) and water (control white). * P < 0.05.

Figure 2 shows the frequency of MNRET in animals exposed by oral administration to compounds **C1–C6** or HU at concentrations of 12.5, 25, 50, and 100 mg/kg. The figure shows that HU, administered at different concentrations (12.5, 25, 50, and 100 mg/kg), induced a significant increase in the frequency of micronuclei in the reticulocytes of mice, relative to that induced by all the synthetic compounds. The average frequencies of MNRET induced by HU at 12.5, 25, 50, and 100 mg/kg were 7.8, 9.8, 15, and 33.7 per 1,000 cells, respectively. This result was not significantly different from that of the positive control, cyclophosphamide (50 mg/kg; i.p.), which induced an average of 46 MNRET per 1,000 cells (Figure 3).

Experiments were also performed using HU at concentrations of 200 and 400 mg/kg. Statistical analysis showed no difference in the micronuclei frequencies at doses of 200 mg/kg and 100 mg/kg. At a dose of 400 mg/kg, a reduction in the number of cells was observed, possibly attributable to the toxicity of HU (results not shown). 

Our results show that HU exhibited genotoxic potential in mammalian cells at all the concentrations tested. These results are consistent with a previous study that demonstrated the genotoxicity of HU using a comet assay, although that assay has the disadvantage of not detecting aneuploidy, chromosomal rearrangements, DNA mis-repair, or DNA adducts [10]. Another study related the genotoxicity of HU to its capacity to indirectly generate hydrogen peroxide, probably by the inhibition of catalase-mediated hydrogen peroxide decomposition [11]. Flanagan and co-workers [12] demonstrated that HU had a detectable genotoxic effect on micronucleus production in humans, although the authors did not establish a relationship between this effect and myelodysplastic syndrome or malignancy symptoms. These results, together with other reports in the literature, allow us to state that HU is a genotoxic agent and a presumed trans-species carcinogen.

Compounds **C1–C6** are organic nitrate esters. This organic function is present in drugs widely used as pharmacotherapies, such as anti-anginal medications. Several reports have pointed out that nitric oxide (NO), generated by organic nitrates, can become deleterious with chronic long-term exposure, with mutagenic and/or carcinogenic effects [13,14,15]. There are at least three mechanism by which NO exerts its genotoxic effects: a) the formation of *N*-nitroso compounds, which can alkylate DNA; b) its reaction with primary amino groups, causing their deamination and leading to base conversion; and c) the formation of highly oxidative species, such as ONOO• and HO•, which can cause oxidative damage. Lin and co-workers reported an increase in the formation of micronuclei when sodium nitroprusside was used as a drug with NO donor properties [16].

All the organic nitrate derivatives evaluated (**C1–C6**) presented average MNRET less than that of HU, despite the probable genotoxicty reported for this function. Compound **C1** produced average MNRET of 2.1, 2.5, 3.0 and 3.9 per 1,000 cells at concentrations of 12.5, 25, 50 and 100 mg/kg, respectively; compound **C2** produced average MNRET of 4.0, 4.3, 4.5, and 5.7, respectively; compound **C3** produced average MNRET of 2.5, 4.5, 5.7, and 6.0, respectively; compound **C4** produced average MNRET of 2.9, 4.2, 4.6 and 5.0, respectively; compound **C5** produced average MNRET of 3.2, 4.5, 5.2, and 5.4, respectively; and compound **C6** produced average MNRET of 1.9, 2.6, 2.9, and 4.6, respectively. These results confirm that compounds **C1–C6** are less genotoxic than HU.

Santos and co-workers demonstrated the lack of mutagenicity of compounds **C1–C6** in an *in vitro* study using a *Salmonella*/microsome assay [9]. Interestingly, the authors showed some structural requirements of these phthalimide derivatives and the mutagenic activity of compounds **C1–C6**. However, this structure–mutagenic activity relationship was not observed in the assay described here, which was based on eukaryotic cells. Many compounds that cause gene mutations also cause chromosomal mutations. However, some mutagenic substances act only at the chromosomal level, and it is essential to use cytogenetic tests to assess the genotoxic potential of all compounds. Therefore, is essential to combine both types of experiments to characterize the genotoxicity of compounds. These mutagenicity and genotoxicity evaluations of compounds **C1–C6** allow us to characterize them as candidate drugs that are safer than HU in the treatment of SCD symptoms.

## 3. Experimental 

### 3.1. Chemicals

Hydroxyurea (HU, CAS No. 127-07-1), carboxymethylcellulose (CMC, CAS No. 9066-07-3), tween-20 (HU, CAS No. 9005-64-5), dimethylsulfoxide (DMSO, CAS No. 67-68-5), acridine orange (CAS No. 65-61-2) and cyclophosphamide (CAS No. 75526-90-8) were purchased from Sigma Chemical Co. (St. Louis, MO, USA). 

### 3.2. Preparation of compounds ***C1–C6***

The compounds were prepared by the condensation of amino alcohol derivatives with phthalic anhydride. The alcohol function of the phthalimide derivatives was converted to the final compounds containing nitrate ester functions [7]. Compound **C4** was prepared, as previously described, by the condensation of sulfonyl chloride [7] with hydroxylamine. Compounds **C1–C6** were characterized by ^1^H-NMR and ^13^C-NMR, infrared spectroscopy, and mass spectrometry. Their purity was confirmed by elemental analysis and high-performance liquid chromatography, and in general, was 99% for all compounds.

### 3.3. Animals

Swiss albino mice (25–30 g) were housed at a constant temperature (23 ± 1.8 °C) and humidity (55 ± 5%), under a 12/12 h light cycle, with food and water *ad libitum*. The experiments were conducted during the light phase. The study protocol was approved by the Research Ethics Committee of the School of Pharmaceutical Science, UNESP, Araraquara, São Paulo, Brazil (process 36/2007).

### 3.4. Evaluation of the mutagenicity of HU and the synthesized compounds with a micronucleus test in peripheral blood cells of mice

The doses of HU and synthesized compounds **C1–C6** were evaluated (12.5, 25, 50, and 100 mg/kg) after they were administered to the animals by gavage. For treatment, the animals were divided into groups of 10, which included five males and five females. A positive control group was established, in which the animals were intraperitoneally treated with cyclophosphamide (50 mg/kg). In the negative control group, the mice were administered 0.3 mL of a solution of 1% carboxymethylcellulose (CMC) and 0.2% Tween by gavage. A group in which the animals received only water were also used. The protocol used for this work was described by Hayashi *et al*. [17], and uses laminas pre-stained with acridine orange. After 30 h, the animals were killed to collect their blood. We counted 1,000 reticulocytes per animal and recorded the frequencies of micronucleated cells. After the cytological analysis of the laminas containing samples of peripheral blood from the mice treated with the various drugs, we calculated the average frequencies of cell micronuclei (and the standard deviations) for each treatment group. These results were tested with analysis of variance (ANOVA). When P < 0.05, the average values for the treatments were compared using the Tukey method, calculating the minimum significant difference at α = 0.05.

## 4. Conclusions

The compounds 1,3-dioxo-1,3-dihydro-2*H*-isoindol-2-yl)methyl nitrate (**C1**), (1,3-dioxo-1,3-dihydro-2*H*-isoindol-2-yl)ethyl nitrate (**C2**), 3-(1,3-dioxo-1,3-dihydro-2*H*-isoindol-2-yl)benzyl nitrate (**C3**), 4-(1,3-dioxo-1,3-dihydro-2*H*-isoindol-2-yl)-*N*-hydroxybenzenesulfonamide (**C4**), 4-(1,3-dioxo-1,3-dihydro-2*H*-isoindol-2-yl)benzyl nitrate (**C5**), and 2-[4-(1,3-dioxo-1,3-dihydro-2*H*-isoindol-2-yl)-phenyl]ethyl nitrate (**C6**) were identified as new candidate drugs for the treatment of SCD by evaluating them with the micronucleus test in peripheral blood cells of mice. All the compounds **C1**–**C6** were less genotoxic than HU, with an average MNRET frequency of < 6 per 1,000 cells, whereas HU induced 7.8, 9.8, 15, and 33.7 MNRET per 1,000 cells at concentrations of 12.5, 25, 50, and 100 mg/kg, respectively. These results allow us to characterize these compounds as new drugs that are safer than HU. The discovery of new drugs with greater safety and efficacy than HU is very important for the treatment of SCD. The next step is to study the pre-formulation and pharmacokinetics of these derivatives.
